# Evidence for evolutionary specialization in human limbic structures

**DOI:** 10.3389/fnhum.2014.00277

**Published:** 2014-05-20

**Authors:** Nicole Barger, Kari L. Hanson, Kate Teffer, Natalie M. Schenker-Ahmed, Katerina Semendeferi

**Affiliations:** ^1^Department of Anthropology, University of California San DiegoLa Jolla, CA, USA; ^2^Psychiatry and Behavioral Sciences, MIND Institute, Department of Psychiatry and Behavioral Sciences, University of California DavisSacramento, CA, USA; ^3^Department of Radiology, University of California San DiegoLa Jolla, CA, USA; ^4^Neuroscience Graduate Program, University of California San DiegoLa Jolla, CA, USA

**Keywords:** emotion, human brain evolution, hippocampus, amygdala, frontal cortex, comparative neuroanatomy, ape, hominoid

## Abstract

Increasingly, functional and evolutionary research has highlighted the important contribution emotion processing makes to complex human social cognition. As such, it may be asked whether neural structures involved in emotion processing, commonly referred to as limbic structures, have been impacted in human brain evolution. To address this question, we performed an extensive evolutionary analysis of multiple limbic structures using modern phylogenetic tools. For this analysis, we combined new volumetric data for the hominoid (human and ape) amygdala and 4 amygdaloid nuclei, hippocampus, and striatum, collected using stereological methods in complete histological series, with previously published datasets on the amygdala, orbital and medial frontal cortex, and insula, as well as a non-limbic structure, the dorsal frontal cortex, for contrast. We performed a parallel analysis using large published datasets including many anthropoid species (human, ape, and monkey), but fewer hominoids, for the amygdala and 2 amygdaloid subdivisions, hippocampus, schizocortex, striatum, and septal nuclei. To address evolutionary change, we compared observed human values to values predicted from regressions run through (a) non-human hominoids and (b) non-human anthropoids, assessing phylogenetic influence using phylogenetic generalized least squares regression. Compared with other hominoids, the volumes of the hippocampus, the lateral nucleus of the amygdala, and the orbital frontal cortex were, respectively, 50, 37, and 11% greater in humans than predicted for an ape of human hemisphere volume, while the medial and dorsal frontal cortex were, respectively, 26 and 29% significantly smaller. Compared with other anthropoids, only human values for the striatum fell significantly below predicted values. Overall, the data present support for the idea that regions involved in emotion processing are not necessarily conserved or regressive, but may even be enhanced in recent human evolution.

## Introduction

Emotional behaviors and the neural structures that subserve them have traditionally been regarded as evolutionarily conserved. Historically, a number of structures have been implicated in emotion production and evaluation and have been commonly grouped together under the umbrella of the “limbic system.” While, in its inception, the limbic system concept was informed by experimental and psychiatric data (MacLean, 1949, 1952), it also became intimately tied to theories of brain evolution. Specifically, its originator, Paul MacLean, proposed that the “paleomammalian” limbic system, present in all mammalian brains, was phylogenetically primitive relative to the “neomammalian” neocortex, which appeared most developed in large brained mammals (MacLean, 1967, 1972). Functionally, in humans, the limbic system was purported to serve animalistic, “visceral” functions, while the neocortex was tied to higher intellectual function (MacLean, 1967). However, contemporary neurophysiological data suggest that emotion does contribute critically to higher order cognitive behaviors, like social decision making (Damasio, 1994; Bechara et al., 2000; Bar-On et al., 2003; Rilling et al., 2008) and theory of mind (Corden et al., 2006; Powell et al., 2010). Additionally, neuroscientific research indicates that limbic structures, while critically involved in motivational and emotional behavior, are also integrated into myriad functional systems (Damasio, 1998; Heimer and Van Hoesen, 2006). For example, the hippocampus participates in anxiety regulation and contextual fear conditioning (LeDoux, 2000; Calandreau et al., 2005; Fanselow and Dong, 2010) as well as episodic and spatial memory (Burgess et al., 2002; Fanselow and Dong, 2010). Thus, from a functional and anatomical perspective, it may be unwise to view the limbic system as a wholly separate, isolated, and primitive module that works in parallel to rather than in concert with non-limbic networks subserving advanced cognitive behaviors. It is perhaps not surprising, then, that emotion is becoming increasingly more central to evolutionary theories of human and non-human primate behavior and cognition, especially those focusing on complex social cognitive abilities like cooperation and social problem solving (Hare, 2007; Herrmann et al., 2007, 2011) or social perception and mentalizing (Aureli and Schaffner, 2002; Parr et al., 2005; Barnard et al., 2007; deWaal, 2008; Byrne and Bates, 2010; Dobson and Sherwood, 2011). At the same time, few recent comparative studies have explicitly addressed the evolution of emotion regulating structures from a neuroanatomical perspective. Given an increased interest in the relationship between emotion and complex cognition in the neuroscientific and evolutionary literature, it may be a fitting time to explicitly address the question: are neural structures associated with emotion necessarily de-emphasized in human brain evolution?

**Figure 1:**
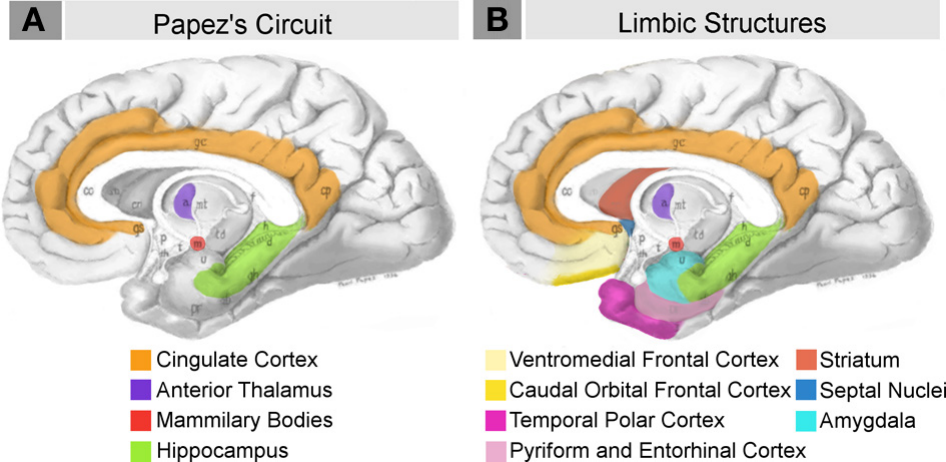
**Schematic briefly summarizing neural systems proposed to process emotion, highlighting structures that are visible on the medial surface of the brain**. Papez's (1937) original circuit **(A)** was expanded upon in the concept of the limbic system **(B)** to include a variety of subcortical and cortical territories (MacLean, 1952; Heimer and Van Hoesen, 2006). (Structures like the anterior insula and nucleus basalis of Meynert, which are not visible on the medial surface of the brain, are not represented here). Images modified from Papez's (1937) original drawing.

A number of neural structures have been hypothesized to underlie emotional expression and evaluation. At the heart of the limbic system is the circuit famously proposed by Papez (1937) as the “anatomical basis of emotion.” It includes the cingulate cortex, anterior thalamic nucleus, hypothalamic mammillary bodies, and hippocampus (Figure 1A). In light of developing experimental evidence, MacLean (1949, 1952) proposed expanding the emotion network to include components of Broca's great limbic lobe (and Turner's rhinencephalon), which Papez's circuit largely overlapped (Figure 1B). As data has subsequently accumulated, the limbic system concept has undergone a number of reformulations and critiques (reviewed extensively in Lautin, 2001). In a recent assessment, Heimer and Van Hoesen (2006) strongly advocate for the anatomical and functional importance of this network, stressing the anatomical contiguity of the structures included in MacLean's original network and their shared contribution to emotional and motivational behaviors. Like MacLean's, their model is based in Broca's great limbic lobe, defined as the region incorporating all non-isocortical pallial structures at the margin of the hemispheres (Figure 1B), i.e., cingulate, anterior insular, pyriform, entorhinal, agranular and dysgranular caudal orbital, ventromedial frontal, and temporal cortex, as well as the hippocampus and corticobasolateral amygdala. They also highlight the importance of regions of the basal forebrain which are functionally and connectively associated with the limbic lobe (Figure 1B), including the striatum, globus pallidus, hippocampus, septal nuclei, hypothalamus, nucleus basalis of Meynert, and extended amygdala (the central and medial amygdaloid nuclei and their extensions into the basal forebrain).

In terms of human brain evolution, only a handful of contemporary volumetric analyses have targeted the evolution of limbic structures. While only a few attempts have been made to perform a broad evolutionary analyses of this system (Vilensky et al., 1982; Stephan, 1983; Armstrong, 1990), some studies have addressed individual structures in this network. In their early volumetric evolutionary analyses, Heinz Stephan and his colleagues included several anatomical constituents of the limbic system in their extensive primate datasets. They reported that these limbic structures are not evolutionarily conserved or regressive by their nature, but that many appeared, “progressive,” i.e., expanded, in humans (Stephan and Andy, 1964; Andy and Stephan, 1968; Stephan, 1983; Stephan et al., 1987). While the methodologies utilized by this group were based on relationships between brain and body size and are not employed in contemporary analyses, on the whole, the few recent volumetric analyses that have addressed this question do not support a systematic reduction of the limbic system in human evolution. Available data suggests that regions of the amygdala incorporated into Heimer and Van Hoesen's limbic lobe may be expanded in humans relative to apes (Barger et al., 2007, 2012) and other primates (Barton and Aggleton, 2000), while the human insula is the size expected for a primate of human brain volume (Semendeferi and Damasio, 2000; Bauernfeind et al., 2013). At the cellular level, the human anterior insula and anterior cingulate cortex are suggested to contain higher numbers of specialized cells relative to apes (Nimchinsky et al., 1999; Hof et al., 2001; Allman et al., 2010), while the chemoarchitecture of the dorsal anterior cingulate (Raghanti et al., 2008a,b,c) and number of neurons in the nucleus basalis of Meynert (Raghanti et al., 2011) do not deviate considerably from other primates. However, few studies have explicitly interrogated the question of limbic system evolution in humans employing phylogenetically informed statistical analyses of volumetric data on multiple structures to investigate more comprehensive changes in this system, as a whole.

To address this issue, we performed an evolutionary analysis of new and previously published data on primate limbic structures. Our goal was to present the most comprehensive volumetric analysis of this system possible with the available data, while highlighting candidate structures for more targeted future analyses. We used assumption free stereological methods to collect new volumetric data on the amygdala and four amygdaloid nuclei, as well as the hippocampus and the striatum, in complete series of histological Nissl stained sections of human and ape brains. We integrated these new data into a novel analysis of previously published hominoid (human and ape) datasets on the amygdala, orbital and medial frontal cortex, and insula, as well as the dorsal frontal cortex, which subserves mostly executive and motor, but not emotional, functions. Additionally, we performed a parallel analysis using published anthropoid data on a large array of anthropoid species (human, ape, and monkey), including the amygdala and two amygdaloid subdivisions, hippocampus, schizocortex, striatum, and septal nuclei. Each dataset was subjected to a novel analysis which: 1. assessed human deviations from allometry by comparing observed human values to values predicted from regressions drawn through multiple non-human primate species, 2. attempted to account for the influence of phylogeny on trait values in individual species in these allometric regressions, and 3. regressed all brain components against the same variable, total hemisphere volume, in order to factor out the influence of increases in brain size on human departures from allometry. Given the increased recognition of the interdependence of emotion and cognition and the central role that emotion may play in human adaptive behaviors, we hypothesized that, in contrast to traditionally held views, individual limbic structures would not necessarily show evidence of decrease in human brain evolution and may even show evidence of human specific specializations.

## Materials and methods

### Materials

**Figure 2:**
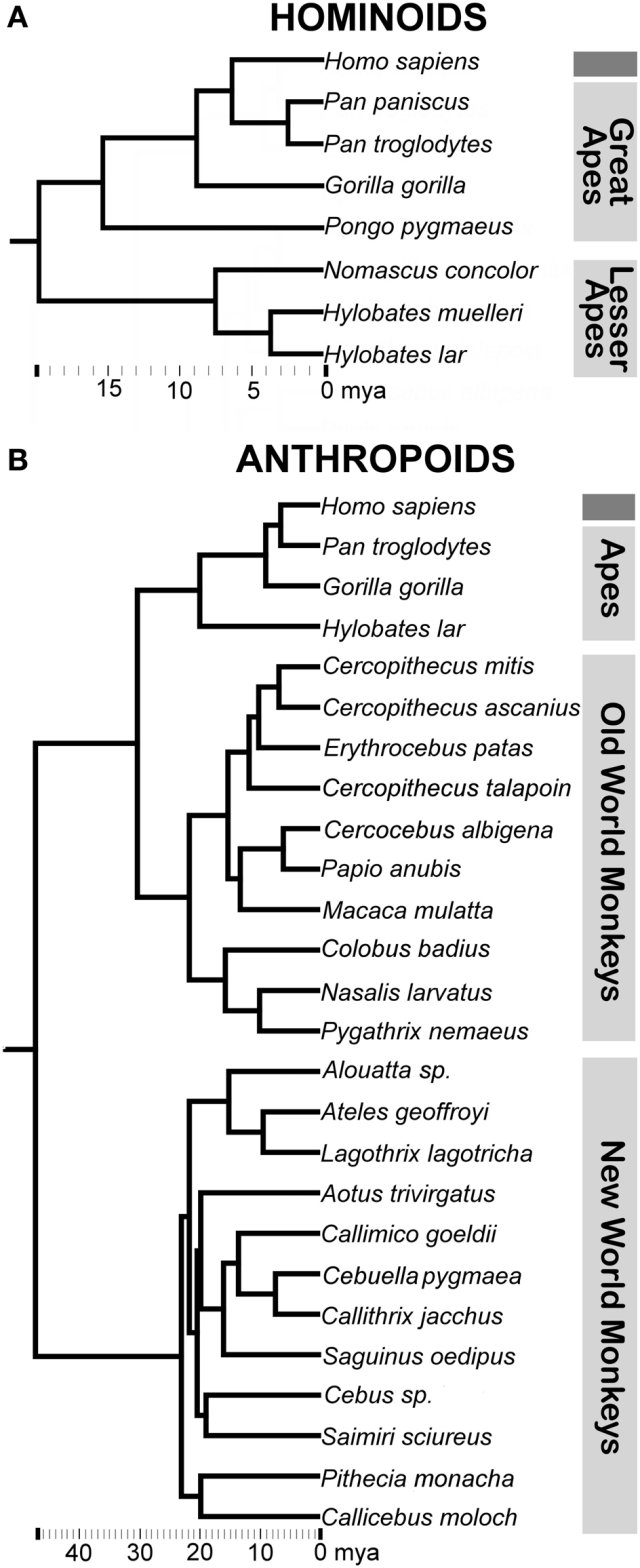
**Consensus phylogeny for (A) hominoids and (B) anthropoids in the analysis with common classifications of major non-human primate clades indicated**. Branch lengths represent evolutionary distance on the scale of millions of years (mya). Consensus phylogenies drawn from the 10 kTrees Website.

Two primary sources, new and previously published data from our own lab (“hominoid datasets”) and the extensive work of Stephan and colleagues (“anthropoid datasets”), provided data suitable for the proposed phylogenetic analyses (Tables 1–3). Specifically, we required that datasets include: (a) volumetric data for multiple limbic structures in addition to hemisphere volume for each individual in the sample in order to ensure the consistency of measures across subjects and (b) more than 4 non-human species to provide sufficient statistical power in regression analyses. Our laboratory's datasets focus heavily on human comparisons with hominoids (apes) (Figure 2A), generally incorporating multiple individuals from each hominoid species (Tables 1, 2). This makes them more suitable for addressing phylogenetically recent patterns of human brain evolution. The Stephan dataset includes a larger array of anthropoid primates (monkeys and apes) (Figure 2B) with one individual data point reported for each species (Table 3). As such, it is appropriate for broader comparisons of human data with trends across anthropoid primates. In apes, the values for three structures, the amygdala, hippocampus, and striatum, are represented in both datasets. Because different methods of data collection were used for each dataset, they were not combined in hominoid analyses to avoid introducing statistical artifacts that may result from these different methodologies. (Including cases from Stephan and colleagues' datasets would only increase our comparatively large hominoid sample by 3 individuals.) For the same reason, we did not incorporate relevant, large datasets that reported on only one primate species (e.g., Freeman et al., 2004; Amunts et al., 2005) or did not include humans (e.g., Sherwood et al., 2004; Carlo et al., 2010).

**Table 1:** **Volume of amygdala, hippocampus, and striatum in hominoid species collected with stereological methods**.

**Species**	**Amygdala**					**Hippocampus**	**Striatum**
	**Lateral**	**Basal**	**Accessory basal**	**Central**	**Total**		
Homo sapiens^*^^,^^a^	0.551^*^	0.452^*^	0.188^*^	0.039	2.031^*^	5.180	9.551
Homo sapiens^*^^,^^a^	–	–	–	–	–	–	12.223
Pan troglodytes^*^^,^^a^	0.138^*^	0.224^*^	0.070^*^	0.065	0.685^*^	1.772	5.159
Pan troglodytes^*^^,^^a^	0.126^*^	0.183^*^	0.070^*^	0.056	0.584^*^	1.520	4.373
*Pan troglodytes*^b^	0.115	0.176	0.066	0.037	0.571	–	–
*Pan troglodytes*^b^	0.095	0.117	0.047	0.024	0.417	–	–
*Pan troglodytes*^b^	0.165	0.263	0.064	0.049	0.749	–	–
Pan paniscus^*^^,^^a^	0.164^*^	0.191^*^	0.066^*^	0.042	0.634^*^	1.710	5.645
Pan paniscus^*^^,^^a^	0.124^*^	0.223^*^	0.070^*^	0.032	0.623^*^	1.595	4.400
Gorilla gorilla^*^^,^^a^	0.100^*^	0.247^*^	0.096^*^	0.037	0.651^*^	1.350	4.626
*Gorilla gorilla*^b^	0.167	0.266	0.112	0.053	0.867	–	–
*Gorilla gorilla*^b^	0.137	0.170	0.105	0.043	0.645	–	–
Pongo pygmaeus^*^^,^^a^	0.124^*^	0.228^*^	0.062^*^	0.047	0.637^*^	1.255	–
Pongo pygmaeus^*^^,^^a^	0.105^*^	0.151^*^	0.052^*^	0.036	0.520^*^	1.415	3.689
*Pongo pygmaeus*^a^	0.157	0.171	0.071	0.030	0.638	–	–
*Pongo pygmaeus*^b^	0.156	0.171	0.067	0.045	0.725	–	–
*Pongo pygmaeus,*^a^	–	–	–	–	–	1.645	4.498
Hylobates lar^*^^,^^a^	0.046^*^	0.063^*^	0.021^*^	0.009	0.203^*^	0.805	1.510
Nomascus concolor^*^^,^^a^	0.060^*^	0.086^*^	0.022^*^	0.012	0.270^*^	0.950	1.994
*Hylobates muelleri*^b^	0.069	0.079	0.030	0.017	0.256	–	–

Data represent one hemisphere in each individual hominoid specimen measured in cubic centimeters (cc). Cases were either:

a paraffin embedded (for processing information see Semendeferi et al., 1998 and Barger et al., 2007) or

b cryosectioned (for processing information see Barger et al., 2012).

Not all cases could be included in all analyses due either to incomplete representation of the region of interest in the series or variation in processing (e.g., sectioning angle) that may affect the precision of anatomical delineations (–). Cases included in Barger et al. (2007) are marked with an ^*^.

**Table 2:** **Volumes of sectors of the frontal cortex (Schenker et al., 2005) and of the insular cortex (Semendeferi and Damasio, 2000) in both hemispheres of individual hominoid specimens measured in cubic centimeters (cc)**.

**Species**	**Frontal cortex**			**Insular cortex**
	**Orbital**	**Medial**	**Dorsal**	
*Homo sapiens*	41.3	68.2	144.6	16.6
*Homo sapiens*	41.3	83.0	179.9	18.8
*Homo sapiens*	33.6	67.3	149.1	14.6
*Homo sapiens*	31.2	66.9	137.7	17.7
*Homo sapiens*	45.6	86.0	174.8	19.0
*Homo sapiens*	44.7	81.7	166.7	18.0
*Homo sapiens*	43.4	90.5	191.5	16.9
*Homo sapiens*	30.0	74.6	185.4	16.5
*Homo sapiens*	35.5	68.9	161.1	15.2
*Homo sapiens*	41.6	67.4	151.3	20.3
*Pan paniscus*	9.0	19.1	35.1	3.5
*Pan paniscus*	13.4	18.2	38.7	4.1
*Pan paniscus*	9.3	17.9	33.3	3.4
*Pan troglodytes*	9.5	15.8	32.6	2.6
*Pan troglodytes*	6.9	12.7	30.3	2.5
*Pan troglodytes*	9.6	15.7	29.6	3.5
*Pan troglodytes*	13.5	22.5	48.4	4.0
*Pan troglodytes*	8.7	17.7	40.5	3.0
*Gorilla gorilla*	11.4	19.7	43.1	3.5
*Gorilla gorilla*	14.7	26.3	52.9	7.1
*Pongo pygmaeus*	9.0	30.1	53.7	5.9
*Pongo pygmaeus*	12.0	33.1	66.5	4.3
*Pongo pygmaeus*	10.8	28.3	65.2	5.3
*Pongo pygmaeus*	7.1	22.7	46.0	3.7
*Hylobates lar*	2.1	3.6	7.7	0.8
*Hylobates lar*	2.4	3.7	7.2	0.7
*Hylobates lar*	2.4	4.2	8.3	0.6

**Table 3:** **Volume of the amygdala (Stephan et al., 1987), hippocampus, schizocortex, septal nuclei, and striatum (Stephan et al., 1981) in anthropoids**.

**Species**	**Amygdala**			**Hippocampus**	**Schizocortex**	**Septal nuclei**	**Striatum**
	**CBL**	**CM**	**Total**				
*Homo sapiens*	1.990	0.653	2.643	5.144	3.071	1.305	14.345
*Pan troglodytes*	0.523	0.188	0.711	1.890	1.009	0.426	6.123
*Gorilla gorilla*	0.999	0.377	1.376	2.391	1.365	0.587	7.284
*Hylobates lar*	0.255	0.078	0.333	1.337	0.568	0.151	2.392
*Papio anubis*	0.370	0.107	0.477	1.699	0.655	0.280	3.591
*Macaca mulatta*	0.247	0.093	0.339	0.677	0.320	0.136	2.016
*Erythrocebus patas*	0.245	0.100	0.344	0.796	0.347	0.165	1.812
*Cercocebus albigena*	0.258	0.133	0.391	0.743	0.315	0.147	2.073
*Cercopithecus ascanius*	0.217	0.070	0.286	0.595	0.347	0.126	1.414
*Cercopithecus mitis*	0.247	0.105	0.352	0.683	0.309	0.123	1.367
*Cercopithecus talapoin*	0.156	0.051	0.207	0.353	0.130	0.067	0.954
*Lagothrix lagotricha*	0.283	0.094	0.377	0.793	0.340	0.133	2.474
*Colobus badius*	0.189	0.062	0.250	0.836	0.407	0.144	1.609
*Pygathrix nemaeus*	0.169	0.076	0.245	1.148	0.362	0.145	1.583
*Nasalis larvatus*	0.250	0.110	0.359	0.983	0.428	0.167	1.868
*Cebuella pygmaea*	0.029	0.009	0.038	0.067	0.041	0.015	0.087
*Callimico goeldii*	0.056	0.018	0.073	0.141	0.069	0.032	0.247
*Cebus* sp.	0.169	0.060	0.229	0.445	0.195	0.087	1.629
*Saimiri sciureus*	0.094	0.027	0.121	0.176	0.084	0.045	0.521
*Pithecia monacha*	0.137	0.046	0.183	0.417	0.145	0.070	0.959
*Alouatta* sp.	0.162	0.051	0.213	0.660	0.255	0.100	1.415
*Ateles geoffroyi*	0.322	0.112	0.434	0.683	0.366	0.162	2.475
*Callithrix jacchus*	0.038	0.015	0.053	0.111	0.045	0.025	0.186
*Saguinus oedipus*	0.055	0.016	0.071	0.131	0.054	0.030	0.227
*Aotus trivirgatus*	0.068	0.028	0.097	0.270	0.122	0.042	0.431
*Callicebus moloch*	0.090	0.037	0.127	0.294	0.117	0.043	0.460

*Data represent one hemisphere in one individual specimen per species measured in cubic centimeters (cc). Abbr: CBL, corticobasolateral division; CM, centromedial division*.

#### Hominoid datasets

Non-human apes in hominoid comparisons included all great ape species (bonobos, chimpanzees, gorillas, and orangutans) and three lesser ape species (Müller's Bornean, lar, and concolor gibbons) (Figure 2A). The following structures were included in our hominoid analyses (Tables 1, 2, Figure 1): 1. amygdala, 2. lateral amygdaloid nucleus, 3. basal amygdaloid nucleus, 4. accessory basal amygdaloid nucleus, 5. central amygdaloid nucleus, 6. hippocampus, 7. striatum, 8. orbital frontal cortex, 9. medial frontal cortex (which was previously defined as including posterior ventromedial limbic cortex and anterior cingulate limbic cortex), 10. insular cortex, and 11. dorsal frontal cortex. Volumes for subcortical structures (items 1–7 above) were collected using serially sectioned post-mortem tissue (for processing details see Barger et al., 2007, 2012). The frontal cortex sectors and the insular cortex (items 8–11 above) are based on structural MRIs and were previously published (Semendeferi and Damasio, 2000; Schenker et al., 2005).

#### Anthropoid datasets

Non-human primates in anthropoid comparisons included multiple species of Old World and New World monkeys and three ape species (Figure 2B) with each species represented by one data point (Table 3). The following structures were analyzed in anthropoid comparisons presented in this analysis (Table 3, Figure 1): 1. amygdala, 2. centromedial amygdala (central, medial, and anterior cortical nuclei and anterior amygdaloid area), 3. corticobasolateral amygdala (lateral, basal, accessory basal, and remaining cortical nuclei) 4. hippocampus (cornu ammonis, dentate gyrus, and subiculum proper), 5. schizocortex (presubiculum, parasubiculum, and enthorhinal cortex), 6. septal nuclei, and 7. striatum. Data for structures other than the amygdala were taken from Stephan et al. (1981). For the amygdala and its subdivisions (items 1, 2, and 3 above), we used the more extensive dataset from Stephan et al. (1987).

### Methods

#### Data collection

Volumes were estimated using the Cavalieri estimator, a stereological technique, in the StereoInvestigator program (MBF Bioscience). Tissue processing and stereological workstation set up has been previous published as part of an initial study on amygdala volumes based on a smaller sample (Barger et al., 2007) and neuronal counts on a large sample (Barger et al., 2012). Stereological parameters for new data on the amygdala, striatum, and hippocampus are presented in Table 4. Nicole Barger collected data for the amygdala, Kate Teffer collected data for the hippocampus, and Kari L. Hanson collected data for the striatum. Briefly, we defined the regions as follows. The amygdala is a heterogenous structure in the medial temporal lobe comprised of 13 discrete nuclei. We present data for four of these nuclei, the lateral, basal, accessory basal, and central nuclei, as well as the whole amygdala as previously defined (Barger et al., 2007, 2012). Following contemporary anatomists and prior comparative work on humans and macaques (Rosene and Van Hoesen, 1987; Duvernoy, 1998), the hippocampus was defined as including the cornu ammonis, dentate gyrus, prosubiculum, subiculum proper, and presubiculum. For improved consistency in delineating the hippocampus from the surrounding entorhinal cortex in diverse species, the very clear boundary between the presubiculum and parasubiculum was used as the endpoint. The striatum was defined as including the caudate nucleus and putamen (Graybiel and Ragsdale, 1978, 1983) to the exclusion of the internal capsule, and the ventral striatum, which includes the nucleus accumbens, olfactory tubercle, and most ventral portions of the caudate and putamen (Heimer and Wilson, 1975; Haber et al., 1990; Holt et al., 1997).

**Table 4:** **Stereological parameters for the Cavalieri estimator used on new cases for the amygdala, striatum, and hippocampus**.

**Stereological parameters for volumetric measurements**	**Amygdala**					**Striatum**	**Hippocampus**
	**Lateral**	**Basal**	**Accessory basal**	**Central**	**Total**		
*Mean # of sections*	10	10	10	9	12	14	16
*Section cut thickness (μm)*	20^a^, 40 – 50^b^	20^a^, 40 – 50^b^	20^a^, 40 – 50^b^	20^a^, 40 – 50^b^	20^a^, 40 – 50^b^	20^a^	20^a^
*Mean # of points counted*	3169	4252	2261	936	12,900	11,653	29,591
*Mean distance between dections (μm)*	788	788	788	788	788	1900	1290
*Grid size for cavalieri (μm^2^)*	150	150	150	150	150	333	150
*Mean coefficient of error (Gundersen m = 1)*	0.008	0.007	0.011	0.016	0.008	0.005	0.001

Nissl stained coronal series were either:

a paraffin embedded (for processing information see Semendeferi et al., 1998 and Barger et al., 2007) or

b *cryosectioned (for processing information see Barger et al., 2012)*.

#### Data analysis

We applied phylogenetic statistics to address the question: Are values for human limbic structures greater than predicted based on values available for: (1) other hominoids and (2) other anthropoids? Consensus phylogenies for hominoids and anthropoids were obtained from the 10 kTrees website (Arnold et al., 2010) and are displayed graphically in Figure 2. In all cases, the species mean value for the neural structure of interest served as the dependent variable. Species mean cerebral hemisphere volume, defined here as the telencephalon and the diencephalon (anthropoid data from Stephan et al., 1981), was used as the independent variable. We chose this measure principally because we were interested in the relationship between gross cerebral expansion and limbic structure volume, but also because other standard measures, like medulla volume, are not consistently available across all datasets. In the analysis, the dependent variable, limbic structure volume, was subtracted from the independent variable, hemisphere volume, to avoid errors that may result from regressing a structure against itself. Because data for both hemispheres were not available for all subcortical structures, volumes for these structures represent the value for one hemisphere. When volumes of subcortical structures were reported in the literature as the sum of structures in both hemispheres, we halved the published values for the sake of measurement consistency. In contrast, volumes for cortical territories are presented as the sum of structure volumes in both hemispheres. Cortical asymmetries were not a focus of this analysis, therefore we did not address this issue, here, but recognize this as a potentially important avenue for future research. This novel analysis provides a consistent analytical framework for all datasets by incorporating a phylogenetic statistical analysis using total cerebral hemisphere volume as an independent variable, allowing us to directly compare results across a wide array of structures.

Data were first subjected to phylogenetic generalized least squares (PGLS) regression analysis using the CAPER (V.0.4) (Orme et al., 2013) module in R (R Development Core Team, 2012) to determine whether phylogeny significantly influenced regressions. Maximum likelihood values for lambda (ʎ) were not significantly different from either 1 or 0 for any structure analyzed. To determine whether human values were significantly greater than predicted, we performed several subsequent analyses. Datasets which indicated no phylogenetic bias (ʎ = 0) were analyzed in SPSS 17 (SPSS, Inc.) using standard least squares regressions. Datasets which yielded evolutionary rates similar to Brownian motion (ʎ = 1) were analyzed using the PDAP module (Midford et al., 2003) of MESQUITE (Maddison and Maddison, 2010) producing least squares regressions with 95% prediction intervals based on independent contrasts. In independent contrasts analysis, branch lengths were not transformed, as contrasts were not significantly correlated with branch length. Regression lines and 95% prediction intervals were procured from PDAP and then mapped back into the original data space to analyze human deviations from predicted values.

For subcortical data, human residuals were considered significantly different if they fell above or below the prediction interval derived from non-human primate data. For cortical data, enough human data points were available to test average residual deviations from values predicted by the regression equation. In SPSS 17, we tested whether predicted values for humans deviated significantly from observed values using a two-sided student's *t*-tests. This was particularly advantageous in the case of the dorsal and orbital frontal cortex, which produced lambda values near 1 in PGLS, necessitating the use of independent contrasts in regression analyses. The PDAP software program for independent contrasts regressions produces somewhat inflated prediction intervals (Midford et al., 2003), which may increase the likelihood of producing a type II error. Calculating the significance of average residual deviations provides a more sensitive, alternative measure testing for significant deviation in independent contrast analyses.

In all cases, percent residual deviations from predicted values were also computed for each structure and are reported to provide for more intuitive interpretation of the results. Percent residual deviations were calculated using non-log-transformed values, i.e., log-transformed values derived from regression equations were inverted prior to calculating the percent. Percent residuals were calculated using a standard approach (see for example Sherwood et al., 2006) by first subtracting the mean predicted value (*Y*_pred_) from the mean observed value (*Y*_obs_), subsequently dividing this number by the observed value (*Y*_obs_), and multiplying the quotient by 100: [(*Y*_*obs*_ − *Y*_*pred*_)/*Y*_*obs*_] * 100.

## Results

### Regressions

Results of regression analyses are summarized in Table 5. The maximum likelihood value of lambda (ʎ) for most structures was not significantly different from 0. Lambda values for the orbital and dorsal frontal cortex were not significantly different from 1. In no case did CAPER yield lambda values that were intermediate between 0 and 1, indicating that the structures either did not show evidence of phylogenetic bias (based on an ultrametric tree) or that evolutionary change was explained by a basic Brownian model, respectively. For all structures, regressions against hemisphere volume were significant at *p* < 0.01. *R*^2^-values were high, ranging from 0.85–0.99, but tended to exceed 0.9 (Table 5).

**Table 5:** **Results of regression analyses run through hominoid and anthropoid values**.

	**ʎ**	**Regression equation**	***R*^**2**^**	***P*-Value**	**95% Prediction interval**	**Human residual (%)**
**HOMINOIDS**						
Amygdala	0	*y* = 0.78x−1.87	0.99	<0.001	0.23 – 0.33	7
A. Lateral nucleus	0	*y* = 0.68x−2.34	0.97	<0.001	−0.56 – −0.35	37^*^
B. Basal nucleus	0	*y* = 0.78x−2.37	0.98	<0.001	−0.33 – −0.13	−30^*^
C. Accessory basal nucleus	0	*y* = 0.88x−3.04	0.91	<0.001	−0.84 – −0.38	−31
D. Central nucleus	0	*y* = 0.98x−3.49	0.95	<0.001	−0.98 – −0.61	−312^*^
Hippocampus	0	*y* = 0.40x−0.69	0.85	0.01	0.27 – 0.56	50^*^
Striatum	0	*y* = 0.74x−0.95	0.97	<0.001	0.91 – 1.25	−27
Orbital frontal cortex	1	*y* = 0.93x−1.32	0.92	0.01	1.07 – 1.99	11^*^
Medial frontal cortex	0	*y* = 1.12x−1.46	0.99	<0.001	1.66 – 2.24	−26^*^
Insular cortex	0	*y* = 1.14x−2.26	0.98	<0.001	1.13 – 1.40	−9
Dorsal frontal cortex	1	*y* = 1.17x−1.20	0.99	<0.001	2.14 – 2.51	−29^*^
**ANTHROPOIDS**						
Amygdala	0	*y* = 0.69x−1.77	0.97	<0.001	0.19 – 0.46	20
A. Corticobasolateral	0	*y* = 0.68x−1.90	0.97	<0.001	0.05 – 0.32	23
B. Centromedial	0	*y* = 0.71x−2.39	0.95	<0.001	−0.42 – −0.03	9
Septal nuclei	0	*y* = 0.74x−2.22	0.98	<0.001	−0.09 – 0.14	19
Hippocampus	0	*y* = 0.75x−1.51	0.93	<0.001	0.53 – 1.03	−17
Schizocortex	0	*y* = 0.77x−1.89	0.95	<0.001	0.23 – 0.66	10
Striatum	0	*y* = 0.91x−1.41	0.98	<0.001	1.20 – 1.49	−54^*^

* *Significant (p < 0.05) human deviations from predicted values based on prediction intervals, for subcortical structures, and student's t-tests on mean human values, for cortical structures*.

### Human residuals for subcortical structures

**Figure 3:**
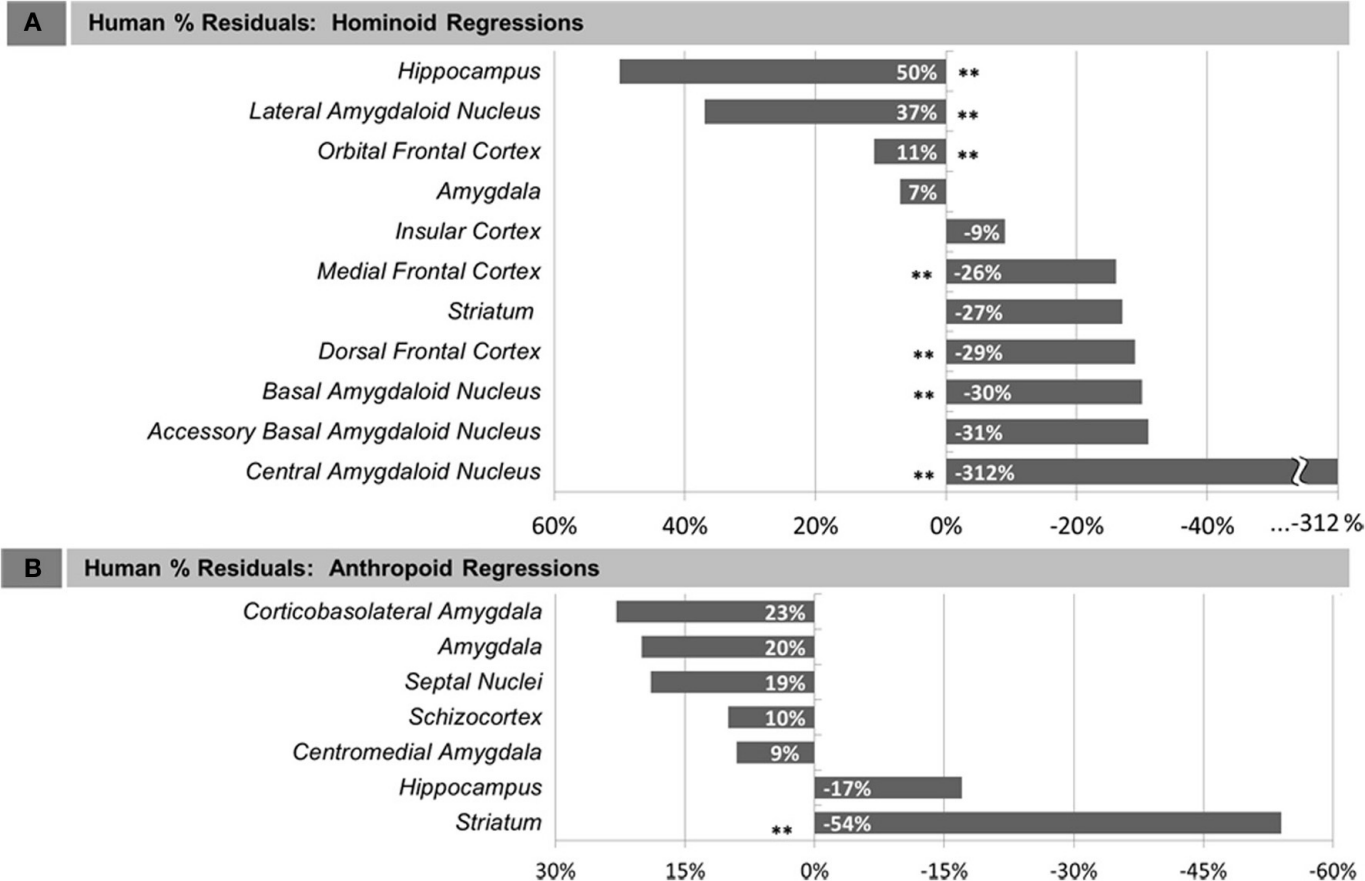
**Human percent residual deviations from non-human regression lines for: (A) Human comparisons with other hominoids and (B) human comparisons with other anthropoids**. Asterisks (^**^) indicate residuals that were significant, determined either by 95% prediction intervals (subcortical structures) or comparison of means at *p* < 0.05 (cortical structures).

**Figure 4:**
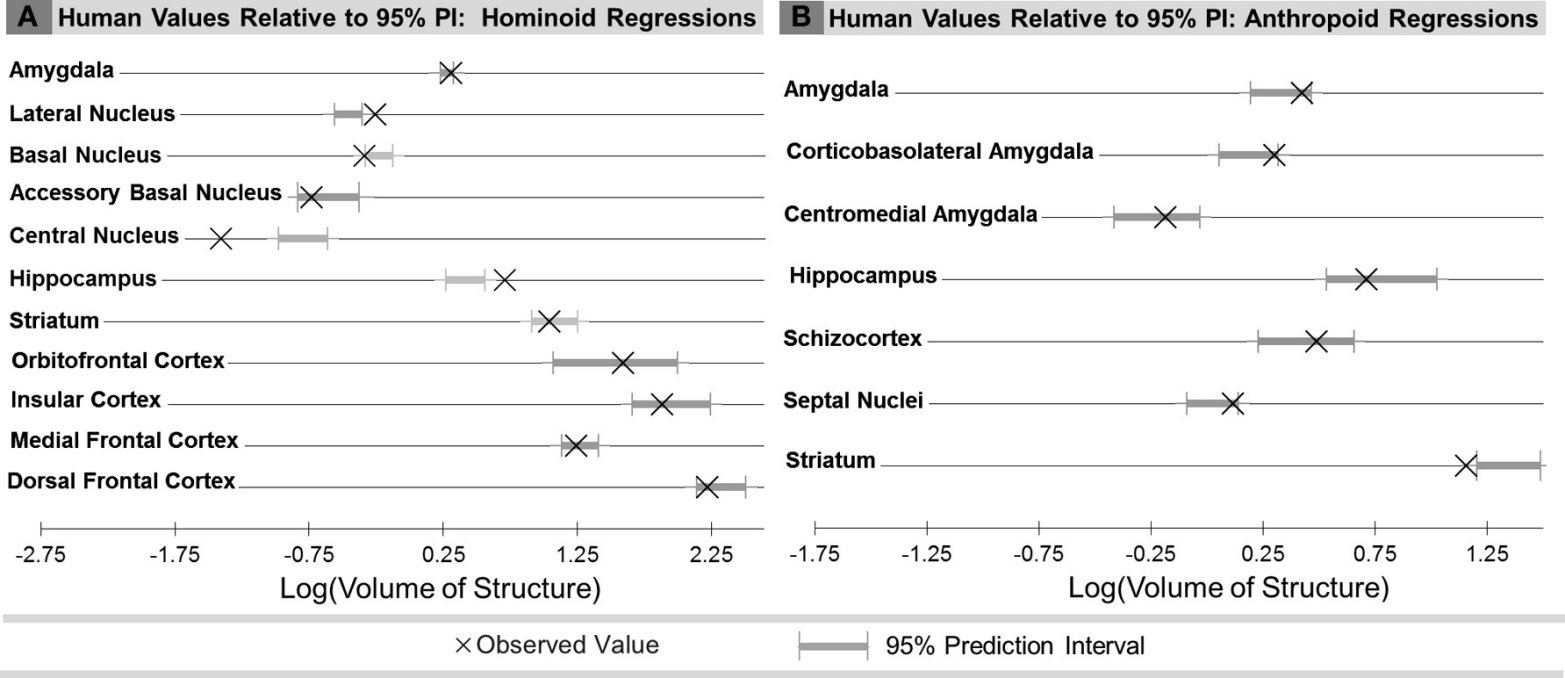
**Comparison of observed human brain structure volumes with the range of values predicted for human hemisphere volume from the 95% prediction intervals (PI) for: (A) Human comparisons with other hominoids and (B) human comparisons with other anthropoids**.

**Figure 5:**
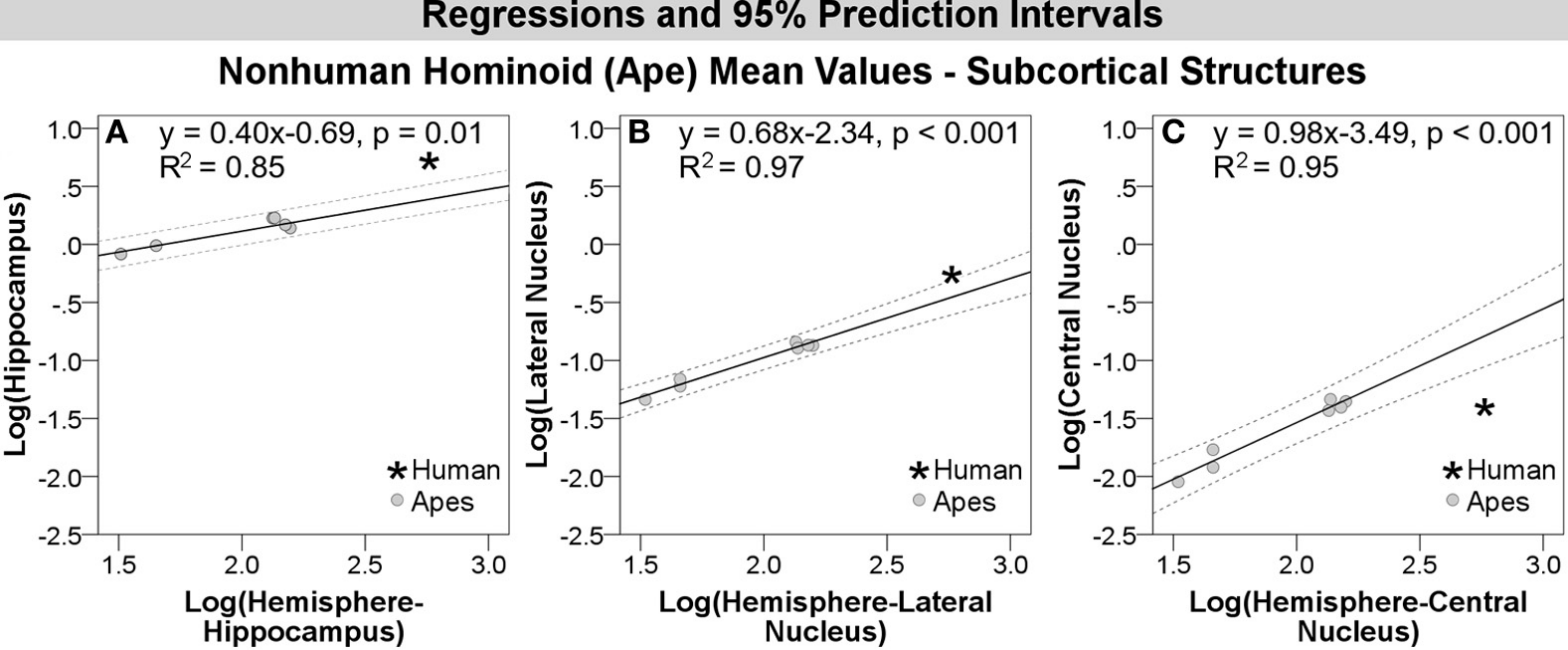
**Log-log regressions and 95% prediction intervals (dashed lines) of species average volumes (cc) for select subcortical structures in the hominoid dataset, which reached significance in statistical analyses, including: (A) hippocampus, (B) lateral amygdaloid nucleus, and (C) central amygdaloid nucleus**.

Compared with other hominoids in analyses of subcortical structures, humans exhibited positive residuals for the hippocampus, lateral amygdaloid nucleus, and whole amygdala from most to least positive (Table 5, Figure 3A). The human residual for the hippocampus fell 50% above the predicted value and outside of the 95% prediction interval (Figures 3A, 4A, 5A). In the amygdala, the value for the human lateral nucleus fell 37% above predicted values (Figures 3A, 4A, 5B). The human amygdala fell modestly (7%) above predicted values and within the upper 5% of predicted values (Figures 3A, 4A). The human observed value was 0.31 (2.03, untransformed); the upper limit of the prediction interval was 0.33 (2.14, untransformed).

Compared with other hominoids, humans exhibited negative residuals for subcortical structures including the striatum, basal amygdaloid nucleus, accessory basal amygdaloid nucleus, and the central amygdaloid nucleus from least to most negative (Table 5, Figure 3A). The values for two structures fell below the prediction interval; the central nucleus fell 312% significantly below predicted values (Figures 3A, 4A, 5C) and the human basal nucleus fell 30% below and just outside of the prediction interval (Figures 3A, 4A).

**Figure 6:**
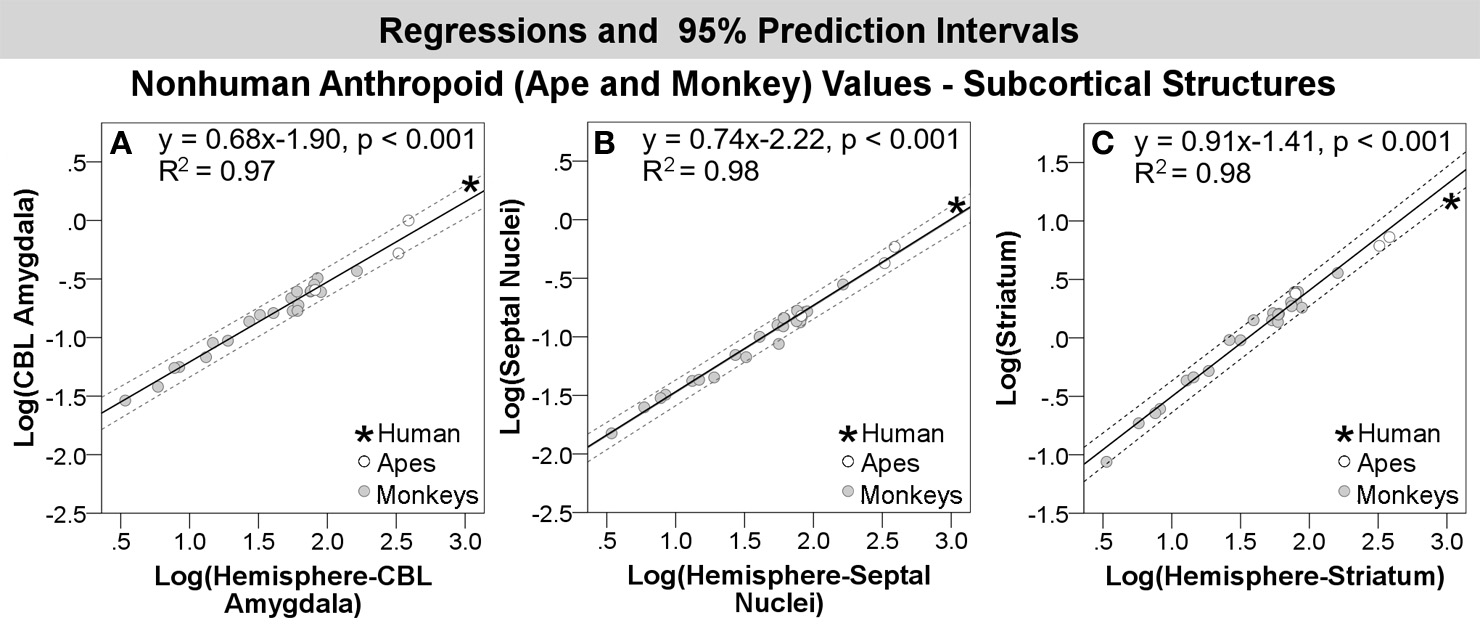
**Log-log regressions and 95% prediction intervals (dashed lines) of species average volumes (cc) for select structures in the anthropoid dataset, which reached significance or fell in the upper bounds of the prediction interval, including: (A) corticobasolateral amygdala, (B) septal nuclei, and (C) striatum**. Abbr: CBL, corticobasolateral amygdala.

Compared with other anthropoids, humans exhibited positive residuals for subcortical structures including the corticobasolateral amygdala, whole amygdala, septal nuclei, schizocortex, and centromedial amygdala from most to least positive (Table 5, Figure 3B). None of these structures fell significantly above the prediction interval (Table 5; Figure 4B), although the corticobasolateral amygdala (Figures 4B, 6A) and septal nuclei (Figures 4B, 6B) fell within the upper 5% of predicted values. For the septal nuclei, the upper limit of the prediction interval was 0.14 (1.38 cc, untransformed) the observed value was 0.11 (1.31 cc, untransformed). For the corticobasolateral amygdala, the upper limit of the prediction interval was 0.32 (2.09 cc, untransformed) and the observed value was 0.29 (1.99 cc, untransformed).

Compared with other anthropoids, humans exhibited negative residuals for the hippocampus and striatum from least to most negative (Table 5, Figure 3B). Only the human striatum fell outside of predicted values, falling below the lower prediction interval (Figures 4B, 6C).

Summarizing across taxonomic groups, humans exhibited positive residuals for the amygdala and several components of the amygdala in all comparisons, significantly positive residuals for the hippocampus in hominoid comparisons and non-significant negative residuals in anthropoid comparisons, and negative residuals for the striatum in both hominoid and anthropoid comparisons, which were only significant in the anthropoid analysis. Other regions were not comparable across groups because they were included in only one of the datasets.

### Human residuals for cortical structures

**Figure 7:**
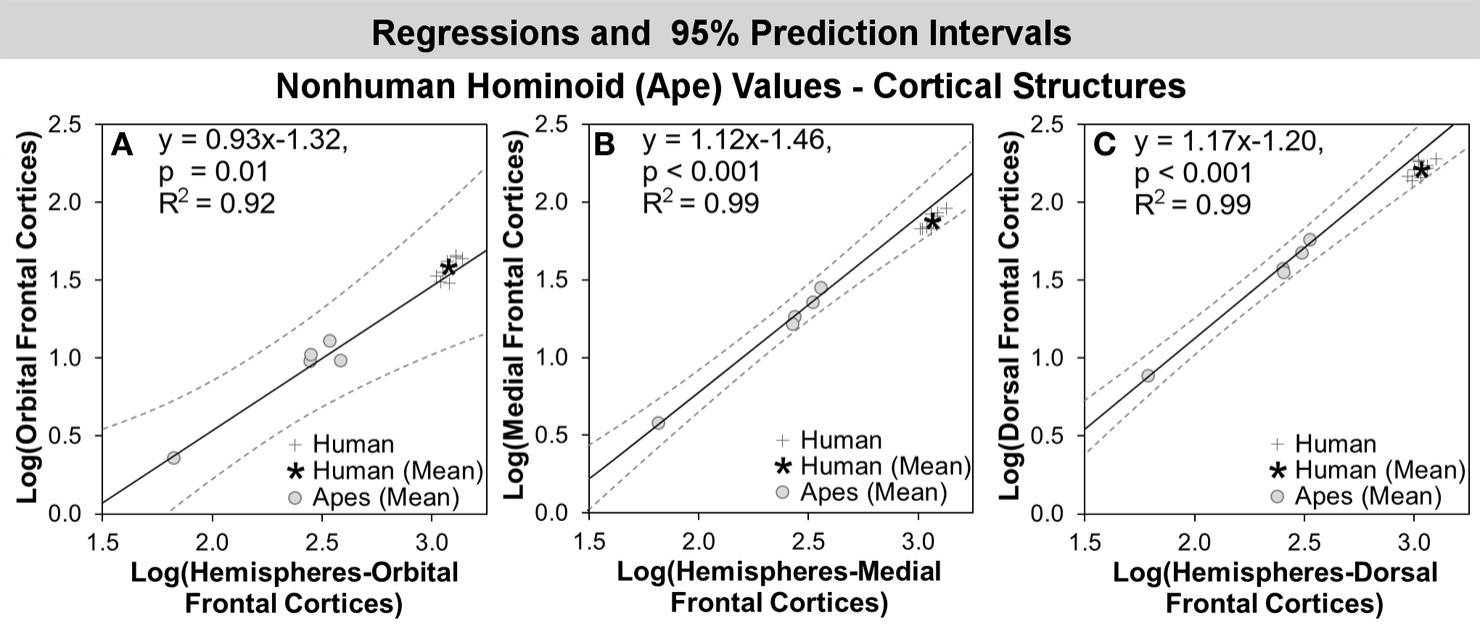
**Log-log regressions and 95% prediction intervals (dashed lines) of species average volumes (cc) for select cortical structures in the hominoid dataset, which reached significance in statistical analyses, including: (A) orbital frontal cortex, (B) medial frontal cortex, and (C) dorsal frontal cortex**.

In comparisons of cortical structures in hominoids, mean human values fell within the prediction interval and only the mean residual for the orbital frontal cortex was positive (Table 5, Figures 3A, 4A). However, in *t*-tests, the mean residual for the human orbital frontal cortex was 11% significantly greater than predicted (*t* = 3.27; *p* = 0.01) (Figure 7A). Orangutan residuals for the orbital frontal cortex were significantly lower than residuals for any other species, in this analysis, suggesting they may be a statistical outlier [One-Way ANOVA: *F*_(6)_ = 6.04, *p* < 0.01; Tukey HSD, *p* < 0.05 for comparisons of orangutans and all other ape species]. Thus, we also ran the independent contrasts regression omitting this taxon (*b* = 1.06; *R*^2^ = 0.98; *p* = 0.01). In this case, the human residual was significantly negative and fell 25% below the prediction interval (*t* = 5.95, *p* < 0.001).

Humans exhibited negative mean residuals for the insula, medial frontal cortex, and dorsal frontal cortex from least to most negative, but all structures fell within the prediction interval (Table 5, Figures 3A, 4A). In *t*-tests, the mean human value for the medial frontal cortex fell 26% significantly below predicted values (*t* = −8.58; *p* < 0.001) (Figure 7B) and for the dorsal frontal cortex fell 29% significantly below predicted values (*t* = −11.01; *p* < 0.001) (Figure 7C). The mean observed value of the insula did not differ significantly from predicted values.

## Discussion

Analyzing new and previously published data, this study presents a comprehensive survey of primate limbic structures with two primary evolutionary questions. First, we wanted to assess whether limbic structures appeared specifically diminished over the course of human evolution, which may be presumed if emotion contributes minimally to advanced human adaptive behaviors. We found that only two of the nine whole limbic structures analyzed across analyses fell significantly below predicted values, the striatum in anthropoid comparisons and the medial frontal cortex in hominoid comparisons. As such, it does not appear to be the case that structures participating in emotion processing are necessarily decreased or de-emphasized in human evolution. Indeed, residuals for many limbic structures exceeded that of the dorsal frontal cortex, a non-limbic region associated with executive and motor function. Given these findings, it can alternatively be asked whether humans show specializations in any limbic structures, consistent with theories emphasizing a greater role for emotional regulation in adaptive human behavior. We found the strongest evidence in favor of human specific adaptations in two subcortical limbic structures, the hippocampus, and amygdala. Additionally, we found that humans may exhibit a modest expansion of the orbital frontal cortex. These distinctions are most apparent when comparisons are made with our closest living relatives, the apes, rather than with a broader array of anthropoids, supporting a model in which derived limbic characters appeared in recent human brain evolution.

### Are human volumes consistent with trends across primates?

#### Human comparisons with anthropoids

When considering trends across a large number of anthropoid primate species (apes and monkeys), human values fell predominantly as expected. In relation to anthropoid primates, the human value for only one structure, the striatum, fell outside predicted values; it was 54% smaller than predicted. The amygdala and its subcomponents, as well as hippocampus, schizocortex, and septal nuclei all fell within the range of predicted values. Although none of the structures in the analysis exceeded the upper prediction interval, the septal nuclei and corticobasolateral amygdala both were approximately 20% larger than predicted and fell within the upper 5% of predicted values, nearly falling outside of the prediction interval (Table 5, Figures 4B, 6). Given these datasets do not present information on the range of volumetric variation in humans (as datasets from Stephan and colleagues include only one human datum), these near significant findings may merit more targeted investigation with larger human samples. In our human and ape comparisons, we have found significant changes in the size of components of the corticobasolateral division. However, little is known about the evolution of the septal nuclei in human and non-human primates.

#### Human comparisons with hominoids

While addressing trends across an array of primate species is instructive, analyzing only hominoids (apes), the group with which humans share the closest phylogenetic relationship, can provide a more pointed assessment of derived neural features occurring in recent human brain evolution at the critical juncture when the human and ape lineages split. Perhaps unexpectedly given their closer phylogenetic relationships, human values for subcortical structures evidenced more significant deviations in comparisons with non-human hominoids than with comparisons across anthropoid species (Figure 3, Table 5). The volume of the human hippocampus significantly exceeded predicted values by 50%. In the amygdala, one amygdaloid nucleus, the lateral nucleus, was significantly increased and had the second highest residual of any structure tested (37%), while other amygdaloid nuclei were significantly smaller than predicted, including the basal (−30%) and central nuclei (−312%). The volume of the whole human amygdala fell in the very upper limits of the prediction interval, as in anthropoids, but only slightly (7%) above predicted values. The volume for the human striatum fell lower than predicted, but within the prediction interval.

We found less evidence for expansion in limbic cortical structures. All cortical structures fell within the predicted range and only the mean residual for the orbital frontal cortex was positive. However, because cortical samples contained multiple human cases, we were able to statistically test whether the mean observed human value deviated significantly from the value predicted by the ape data. Using that measure, mean human orbital frontal cortex volume appeared modestly and significantly larger than predicted by the regression analysis (11%). In contrast, the mean volume of the medial frontal cortex was significantly smaller than predicted (−26%). The insula fell very close to predicted values (−9%), within the prediction interval, and a *t*-test for differences in the mean residual was not significant, providing robust evidence that the human insula is approximately the size expected for an ape of human hemisphere volume.

The mean volume of the dorsal frontal cortex, a non-limbic cortical territory included for comparison, similarly, did not appear expanded in humans. In fact, among cortical territories, it exhibited the most negative mean residual (−29%). The mean human data point was contained within the prediction interval but was significantly different from values predicted by the regression equation. The dorsal frontal cortex is largely engaged in executive and motor function (Fuster, 2001). Because it is a functionally heterogeneous region, it is unwise to make explicit statements contrasting the importance of emotion and executive or motor function in human brain evolution. However, the fact that volumes in this region are, on average, smaller than predicted in humans does lend further credence to the idea that the network of structures involved in emotion processing are not especially reduced relative to what would be expected based on evolutionary trends in other functionally distinct regions.

#### Comparisons between hominoids and anthropoids

Although the structures reported in our ape dataset and Stephan's anthropoid dataset do not entirely overlap, three structures, the hippocampus, amygdala, and striatum, are present in both datasets. This allows us to contrast evolutionary findings for these structures in anthropoids and hominoids, directly. It is important to reiterate that, across studies, slightly different regions were analyzed, and different methodologies were used. Because combining the two datasets may introduce artifacts related to these methodological differences, we chose to include only data that were collected in a consistent manner in each regression analysis. Of course, when comparing across analyses, it is important to address the fact that some hominoids are included in the anthropoid regressions, potentially compromising phylogenetic comparisons between the two taxa. However, removing hominoids from the anthropoid regression minimally influences the slope in all cases (Hippocampus: slope = 0.78 without hominoids, slope = 0.75 with hominoids; Striatum: slope = 0.94 without hominoids, slope = 0.91 with hominoids; Amygdala: slope = 0.69 without hominoids, slope = 0.69 with hominoids). Equally, running a regression through the 3 ape data points in the Stephan sample produced a slope comparable to the slope run through our expanded ape dataset (Hippocampus: slope = 0.32 with Stephan data, slope = 0.40 with our data; Striatum: slope = 0.69, with Stephan data, slope = 0.74 with our data; Amygdala: slope = 0.77 with Stephan data, slope = 0.78 with our data), although these analyses did not reach significance likely due to low statistical power. Methodologically, concordant findings between analyses run on individuals in our dataset, who spent much of their life in zoos, and individuals in the Stephan dataset, who were largely wild caught, provide some assurance that the results of our human and ape comparisons are not simply artifacts of neural responses to captive environments.

Differences in the degree and direction of human residuals produced in anthropoid and hominoid analyses may reflect variation in allometric scaling. The residual for the hippocampus fell significantly above the regression line only in comparisons with hominoids, while the striatum fell below the regression line only in anthropoid comparisons. In hominoids, the slope for hippocampal volume was 0.40, while in anthropoids, it was nearly doubled at 0.76. Similarly, the coefficient for the striatum was lower in hominoids, 0.74, than in anthropoids, where it nearly reached isometry at 0.91. Concordantly, human data points for the hippocampus fell considerably more positively and for the striatum somewhat more positively in hominoid comparisons than in anthropoid comparisons. In contrast, the scaling coefficient for the amygdala was higher in hominoids, 0.78, than in anthropoids, 0.60, and the positive human amygdala residual was less substantial in the hominoid analysis. A slope, or allometric scaling coefficient, of 1 would indicate that a structure is increasing at the same rate as the total size of the hemispheres in a particular taxon. Higher coefficient for the amygdala in hominoids suggests that volumetric increases in the hemispheres and amygdala are more tightly coupled in hominoids than across their parent taxonomic group, anthropoids, which evidence a much tighter relationship between hemisphere and striatum volume. It may even be the case that great apes have reduced striatum volumes, as the chimpanzee and gorilla data points fell considerably below the regression line in anthropoid analyses whether they were included (Figure 6C) or excluded from the regression. However, the individual points were within the prediction interval. Amygdala and striatum scaling coefficients were approximately twice the size of the hippocampus coefficient in hominoids, suggesting that increases in hemisphere volume are more tied to increases in amygdala and striatum volume rather than hippocampal volume in this taxonomic group.

In anthropoids, the corticobasolateral amygdala exhibited the highest residual and, in apes, we assessed three individual amygdaloid nuclei that comprise the majority of the corticobasolateral amygdala. The largest nucleus in the human corticobasolateral division is the lateral nucleus and the human residual for this nucleus was significantly and substantially positive (*n.b*. The Stephan group switched terminologies for the corticobasolateral division from “corticobasolateral amygdala” (Stephan and Andy, 1977) to “lateral amygdala” (Stephan et al., 1987). The lateral nucleus is a constituent of and not synonymous with Stephan and colleagues' “lateral amygdala.”). Two other nuclei in the corticobasolateral group, the accessory basal and basal nuclei, were not similarly increased in humans. As such, it is tempting to speculate that the expansion of the lateral nucleus may drive the high residual for the human corticobasolateral division.

### Comparisons with previous analyses

#### Anthropoids

Stephan and colleagues have previously reported that limbic structures are expansive in humans using this dataset. However, these findings have often been difficult to reconcile with contemporary evolutionary analyses due to methodological differences. In an effort to improve upon previous studies that used proportional measures, Stephan and colleagues developed the “progression index” (Stephan and Andy, 1977; Stephan, 1983). This allometrically based measure assessed how much larger, or more “progressive,” a neural structure was in a primate of a certain body size relative to what would be predicted for a “primitive mammal,” i.e., insectivore, of a similar body size. If a neural structure in a primate had a “progression index” of 2, for example, that structure is twice the value expected for an extrapolated basal insectivore of similar body size as the primate species in question. Stephan and colleagues reported that human “progression indices” were positive for all limbic structures and were two to three times greater than in chimpanzees, indicating that they were particularly expansive by this metric. Ordered from most to least “progressive,” human “progression indices” for limbic structures were: striatum, 16 (Stephan and Andy, 1964), corticobasolateral amygdala, 6.2 (Stephan et al., 1987), schizocortex, 5.5 (Stephan, 1983), septal nuclei, 4.5, (Andy and Stephan, 1968), whole amygdala 4.4, (Stephan et al., 1987), hippocampus, 4.2 (Stephan, 1983), and centromedial amygdala, 2.4 (Stephan et al., 1987).

Using contemporary allometric methods, we did not find such extensive evidence of expansion. Only human residuals for the corticobasolateral amygdala and septal nuclei fell near the upper bounds of the prediction interval. Residuals for most structures were well within the prediction interval. However, in contrast to predictions from “progression indices,” the striatum was the only structure to fall significantly below the prediction interval. Discrepancies between our findings and Stephan and colleagues are likely due to brain-body size scaling. Because human brain size is especially large relative to body size and overall brain size is one of the best predictors of brain component size (Finlay and Darlington, 1995), the high human “progression indices” reported for all limbic structures may predominantly reflect human departures from allometric scaling between brain and body size as opposed to adaptive deviations in the size of each individual structure analyzed.

Our findings are generally concordant with more recent analyses of the Stephan datasets. Barton and Aggleton (2000) and Barton et al. (2003) suggest that the corticobasolateral region has been particularly important in human and anthropoid primate evolution. They found that, relative to medulla volume, this region is larger in haplorhine primates (anthropoids and tarsiers) than in strepsirrhine primates (other prosimians), while the relative size of the centromedial division does not appear to differ across clades. Consistent with our findings, the residual for the human datum in their analysis fell substantially above the regression line for the corticobasolateral amygdala, but less so for the centromedial amygdala (Barton and Aggleton, 2000). In contrast, using this dataset, other authors have suggested that the limbic system is reduced in primates, particularly in relation to the neocortex (Finlay and Darlington, 1995; Reep et al., 2007). However, when the limbic factor was investigated in more detail across 112 mammalian species, two components appeared to segregate, an olfactory limbic factor, incorporating the olfactory bulb, paleocortex (including primarily the olfactory cortex), schizocortex, and hippocampus, and a non-olfactory limbic factor, loading heavily on the amygdala and septal nuclei (Reep et al., 2007). Ultimately, the model that evidenced a tradeoff between limbic and neocortical factors in primates defined the limbic system as the olfactory limbic factor plus the septum (Reep et al., 2007). In this light, it is interesting that, using the same primate dataset, we found the highest positive human residuals for constituents of the non-olfactory limbic factor, the amygdala, corticobasolateral amygdala, and septal nuclei. Indeed, other studies have emphasized the strength of amygdalo-cortical connections in primates (Young et al., 1994), while neocortical increase has been linked with concomitant increase, rather than reduction, in the corticobasolateral amygdala, specifically (Barton et al., 2003).

#### Hominoids

Given the considerable expansion of the cortex in human evolution, it may be surprising that a subcortical component of the limbic lobe, the hippocampus, provided the greatest evidence for evolutionary change in humans, appearing 50% larger than predicted. When we drew a regression line through hippocampal data from the apes in Stephan et al. (1981), their human data point fell 40% above predicted values, consistent with our findings. Beyond the work of Stephan and colleagues, there are few comparative primate datasets for the hominoid hippocampus.

We found evidence for evolutionary specializations in the human amygdala and this is in agreement with prior studies which point to the amygdala as a target of evolutionary change. In addition to differential expansion of the corticobasolateral and centromedial amygdala (Barton and Aggleton, 2000), the gross position of the amygdala in the hemispheres is suggested to have shifted in humans, relative to apes, as the human temporal lobe has expanded (Aldridge, 2011). We have confirmed our previous finding that the human lateral nucleus is larger than predicted for an ape of human hemisphere volume (Barger et al., 2007) and have shown that it is among the most expanded limbic structures, second only to the hippocampus. However, we have also found that the human central nucleus is over three times smaller than predicted for an ape of human hemisphere volume, which had not been previously established. Additionally, the basal amygdaloid nucleus appears significantly decreased in this analysis, although it just skirts the lower bounds of the prediction interval. We have recently reported that neuron numbers tend to loosely follow these trends (Barger et al., 2012), with more extreme differences in the lateral nucleus and more moderate decreases in the central and basal nuclei. The human lateral nucleus contains nearly 60% more neurons than predicted for an ape with a similar number of amygdala neurons; neuron numbers in the central nucleus are 12% fewer in humans; and neuron numbers were only 7% decreased in the human basal nucleus, a difference which only approached significance.

Given evidence for more significant decrease than increase in its constituent nuclei, one might expect to see a reduction in the overall size of the human amygdala. This was not the case. Human amygdala volume was within the range of predicted values and fell slightly above the regression line. Gains in the human lateral nucleus appear to compensate for the diminished size of the central nucleus. Because it is such a small structure to begin with, comprising approximately 5–10% of the amygdala in apes (Table 1, Barger et al., 2007) and 2% in humans (Table 1, Schumann and Amaral, 2005; Barger et al., 2007), it may be the case that a three-fold decrease in central nucleus size does not substantially affect overall amygdala volume. In sum, the data suggest that human amygdala evolution is characterized by general conservation in overall size coupled with a substantial increase in the volume and number of neurons in the lateral nucleus and significant volumetric decreases in the central and basal nuclei with slight neuronal decreases in these two nuclei.

From an evolutionary perspective, we and others have argued that specializations in the amygdala are best described by the process of evolutionary reorganization (Barton and Aggleton, 2000; Barger et al., 2007; Semendeferi et al., 2010). That is to say, while the overall size of this structure is not substantially increased, its intrinsic components evidence a different organization than closely related species, which may ultimately have implications for function. Differential volumetric change in nuclei has a consequences for the overall organization of the amygdala in humans and apes. Specifically, in all ape species analyzed, the basal nucleus is the largest amygdaloid nucleus (Barger et al., 2007, Table 1). Concomitant with evolutionary expansion in the human lateral nucleus and decrease in the basal nucleus, the lateral nucleus has become the largest nucleus in the human amygdala (Schumann and Amaral, 2005; Barger et al., 2007, Table 1). Distinct patterns of neuronal morphology and complexity may be of particular importance in primate evolution (Bianchi et al., 2013; Hrvoj-Mihic et al., 2013). Volumetric changes may specifically reflect variation in neuropil which is comprised largely of dendritic branches and axonal fiber tracts. Thus, the human lateral nucleus may gain more cellular and connective resources relative to other amygdaloid nuclei, a case distinct from related species.

Functionally, this sort of reorganization may reflect an evolutionary shift in computational emphasis. The role of the amygdala in emotion processing is highly integrative and it is comprised of many nuclei which have been separated into distinct subgroups based on their chemical, connective, and developmental properties. The lateral, basal, and accessory basal nuclei share strong connections with cortical regions (Stefanacci and Amaral, 2002). Heimer and Van Hoesen (2006) have grouped these nuclei with the cortical constituents of the limbic lobe, while Swanson and Petrovich (1998) argue that the lateral and basal nuclei are likely to represent deep layers of the temporal cortex and are best understood as components of the fronto-limbic system. This is consistent with previous analyses that have found that corticobasolateral amygdala increase correlates with neocortical expansion (Barton et al., 2003). Corticobasolateral increase also correlates with increase in the lateral geniculate nucleus, possibly reflecting greater reliance on visual information processing in primates (Barton and Aggleton, 2000). In contrast, the nuclei of the centromedial division are more aligned with the striatum in the models proposed by Heimer and van Hoesen and Swanson and Petrovich, and centromedial amygdala volume is linked to changes in the striatum and olfactory system in anthropoid primates (Barton et al., 2003; Barton, 2006). Moreover, the central nucleus provides the primary output to brain stem nuclei controlling physiological aspects of emotional response (Freese and Amaral, 2009). Because the lateral nucleus is the primary target of afferent connections arriving from higher order visual and auditory processing centers in the temporal lobe (Stefanacci and Amaral, 2002), we suggest that expansion of the lateral nucleus may be associated with the significant evolutionary expansion reported for the human temporal lobe (Semendeferi and Damasio, 2000; Rilling and Seligman, 2002). It is likely that human lateral nucleus neurons receive and send more information from the temporal lobe, necessitating larger and/or more numerous dendritic branches than central nucleus neurons. In the human amygdala, this suggests that more neural and connective resources are shifted toward the initial evaluation of the emotional salience of cortically derived sensory input, a primary function of the lateral nucleus, and away from the modulation of brainstem nuclei mediating somatic response (Barger et al., 2012).

Compared to the hippocampus and amygdala, residuals for cortical limbic and non-limbic structures were not as extreme nor as positive. This is also consistent with prior analyses. In several studies, we have found that some cortical regions, like the frontal lobe and insula, are not disproportionately increased in human comparisons with apes (Semendeferi and Damasio, 2000; Semendeferi et al., 2002). As such, it is not surprising that residuals for the medial and dorsal frontal cortex were not high in humans, and we further substantiate that the size of the human insula is in the size range expected based on our shared ancestry with apes. In a recently published, stereological analysis based on a broader sampling of primate species, Bauernfeind et al. (2013) also found that the size of the whole human insula fell close to allometric predictions.

The one limbic cortical structure that appeared to stand out in this analysis is the human orbital frontal cortex. It was modestly, but significantly, larger than predicted for an ape of human brain size. Although it is intriguing, we are cautious not to overstate this finding. We did find that the volume of the orangutan orbital frontal cortex is significantly smaller than other species, consistent with our previous analysis (Schenker et al., 2005). If they are treated as an outlier taxon and excluded from the analysis, the mean volume of the human orbital frontal cortex was significantly negative (−25%), with a residual close to that of the medial frontal cortex. As such, we cannot unequivocally say that the human orbital frontal cortex is significantly greater than predicted. It may follow a pattern consistent with the rest of the frontal lobe (Semendeferi et al., 2002). Moreover, in the only stereological evolutionary analysis of a cytoarchitectonic territory in the orbital frontal cortex, we previously found that posterior orbital area 13 was especially reduced in humans and was large in orangutans (Semendeferi et al., 1998). However, the human orbital frontal cortex contains five distinct cytoarchitectonic regions, making it is possible that other regions are expanded, even if orangutans are taken to be a true outlier influencing human deviations from the line (Ongür et al., 2003). More intensive stereological analyses focusing on discrete cytoarchitectonic regions are needed to better understand orbital frontal cortex evolution in the human brain.

As a caveat, it is important to note that, because a primary purpose of this paper was to survey data available in the literature to generate hypotheses for future tests, some structures in the analysis contain both limbic and non-limbic components. This is especially true of the cortical data, which were drawn from MRIs. Consequently, regional boundaries were based on gross landmarks and not individual cytoarchitectonic territories. The medial frontal cortex and insula are functionally and structurally heterogeneous. It may well be the case that limbic subcomponents exhibit different evolutionary trajectories than entire structures in hominoid comparisons. Bauernfeind et al. (2013) found that humans values for the limbic component of the insula did not deviate significantly from predictions, but the authors note that the fold difference in size change between humans and chimpanzees is greater in this region than in any region of the brain previously analyzed, including portions of the prefrontal cortex. In contrast, no complete cytoarchitectonic region of the medial frontal cortex has been parcellated for comparative volumetric analysis. Of course, this caveat is not limited to the limbic cortex. The cytoarchitectonic regions of the dorsal frontal cortex more associated with executive function have yet to be dissociated from the motor and premotor areas for volumetric analysis, making it premature to conclude that dorsal frontal executive structures are more or less expansive relative to orbital limbic structures.

### Functional and evolutionary significance

Human volumes for most of the structures analyzed fell within the range of expected values, providing little evidence for large scale reduction of this network in human evolution. This was especially the case when humans were compared with a broader array of anthropoid primate species. Strikingly, only in human comparisons with other hominoids did any structure actually exceed predicted values. If increases in volume are the result of positive selection on this neural system, the data best support a model in which critical adaptations in the human limbic system arose on a more recent evolutionary timescale, after human and ape lineages split. In particular, the human hippocampus and amygdala appear to be evolutionarily derived compared to what may be expected based on trends in apes, and the orbital frontal cortex may be slightly more emphasized in recent human evolution. Expansion in these structures could have implications for important adaptive behaviors.

Increases in the volume of the amygdala and orbital frontal cortex have been shown to correlate with behavioral variables related to social complexity and cognition, suggesting a tentative link between structure and function. The amygdala and orbital frontal cortex share a long tenure as the central members of the “social brain” system (Kling and Steklis, 1976; Brothers, 1990; Adolphs, 2009). Amygdala volume positively predicts online and real-world social network sizes in humans (Bickart et al., 2011; Kanai and Bahrami, 2012) and rates of social play across primates (Graham, 2010). The corticobasolateral portion of the amygdala, which exhibited the highest residual in anthropoid comparisons and contains the expansive lateral nucleus, scales with social group size across primates (Barton and Aggleton, 2000). The relationship between individual amygdaloid nuclei and social group size has not been assessed. However, in two disorders characterized by aberrant social behavior, autism spectrum disorder (Schumann and Amaral, 2006) and William's Syndrome (Galaburda and Bellugi, 2006), the lateral nucleus appears to be the most affected amygdaloid nucleus and is suggested to contribute significantly to neuropathology. Increased orbital frontal cortex volumes are associated with enhanced performance on intentionality tasks related to higher order social cognition (Powell et al., 2010) and numerous neuroimaging experiments link anterior regions of the orbital frontal cortex to higher order social behavior (Kringelbach and Rolls, 2004). Although these studies do not provide explicit causal links, taken together they suggest that changes in the size of these limbic structures and their subcomponents may be associated with variation in social behavior. Given the association between emotion regulating structures and social complexity, it is tempting to hypothesize that the complexity of human social behaviors and groups precludes comprehensive evolutionary regression of the limbic system.

However, this story is somewhat complicated by the fact that the hippocampus, which appeared to be the most expansive region, is involved in a number of processes that are not explicitly social in nature. For example, the hippocampus is heavily involved in declarative and episodic memory (Bechara et al., 1995; Thompson and Kim, 1996; Fanselow and Dong, 2010) and hippocampal volume correlates with several measures of executive function across primates (Shultz and Dunbar, 2010). Like many structures in the brain, the hippocampus is also functionally heterogeneous. The anterior hippocampus is more integrated into emotion regulating systems and the posterior hippocampus is associated with topographic memory (Fanselow and Dong, 2010). While posterior hippocampal volume may be associated with measures of ecological intelligence, e.g., the well-known findings for London taxi cab drivers (Maguire et al., 2006), total hippocampal volume has not been shown to correlate with measures of ecological intelligence in primates (Barton, 2000). Indeed, in taxi cab drivers, increases in posterior hippocampal volume were accompanied by decreases in the anterior hippocampus, resulting in no overall change in the size of the whole structure. Affective disorders, like depression or post-traumatic stress disorder, have been associated with smaller overall hippocampal volumes (Villarreal et al., 2002; Orme et al., 2013), however comparative measures of socially relevant affect in healthy primate populations are largely unavailable. This leaves emotion's contribution to variation in anterior or total hippocampal volume an open question. From an anatomical perspective, parcellating the anterior and posterior territories for comparative analysis may provide greater insight into the behavioral correlates of hippocampal expansion in human evolution.

The amygdala, anterior hippocampus, and orbital frontal cortex do share some functional attributes that could be argued to underlie coordinated evolutionary changes in these structures. All are heavily involved in circuits underlying implicit learning and memory as well as emotion modulation (Kringelbach and Rolls, 2004; Freese and Amaral, 2009; Fanselow and Dong, 2010). The amygdala and hippocampus subserve cue dependent and context dependent fear conditioning (LeDoux, 2007; Fanselow and Dong, 2010), while the orbital frontal cortex is essential for monitoring reward and punishment values (Kringelbach and Rolls, 2004). Coordinated change in these structures could support theories which hypothesize that changes in neural systems modulating emotional responses to conspecifics and/or emotion regulated learning and memory are key features of human cognitive evolution.

Highly interconnected with the amygdala and hippocampus, the septal nuclei are also involved in memory and sociality. Values for the human septal nuclei almost exceeded the upper limits of the prediction interval and may appear significantly increased in a larger sample. In humans, the septal nuclei are activated in social behaviors like cooperation, emotional attachment to conspecifics, and even attachment to abstract ideologies which may form the foundation of cultural affiliation (Moll and de Oliveira-Souza, 2009). The volume of the human septal region has been shown to correlate with “source memory,” e.g., the ability to recall the source of received information (Butler et al., 2012). Given the important role attributed to memory, affiliation, and emotional processing in human social cognitive evolution (Hare, 2007; Byrne and Bates, 2010), the evolution of the septal nuclei in human and non-human primate brains may be a fruitful object of further study.

In contrast, residuals for the striatum were consistently low, despite its involvement in a variety of important cognitive processes, including learning and memory (Grahn et al., 2009). In this structure, there is evidence to suggest that investigations of species-level differences in processing may be most meaningful at the cellular rather than gross volumetric level. The limbic ventral striatum, and particularly the nucleus accumbens contained therein, serves as an important site for the processing of reward-related stimuli and the reinforcement of motivated behavior via rich innervation of neurotransmitters, such as dopamine (Haber et al., 2006). Variation in the distribution of neuropeptides, such as oxytocin, has been found to correlate with patterns of social organization (Ross et al., 2009). Similarly, important neural dynamics in the accumbens region may play a role in the regulation of behavioral responses to reward stimuli (Zahm, 1999), which seem to vary in species-specific patterns relative to social organization (Amici et al., 2008).

### Conclusion

Given historical perspectives on brain evolution, one might predict that cortical territories, especially the non-limbic dorsal frontal cortex, should be more expansive than limbic subcortical structures in human brain evolution. Strikingly, we found the strongest evidence for limbic structure expansion in subcortical limbic structures and in human comparisons with apes but not with all anthropoids. The size of the human hippocampus was 50% larger and the lateral amygdaloid nucleus nearly 40% larger in humans than predicted for an ape of human hemisphere volume. Humans may also exhibit a modest increase in the size of the orbital frontal cortex. Although this analysis is the most comprehensive assessment of limbic structures in human evolution to date, it is also a preliminary assessment of potentially productive avenues for future research. The septal nuclei may be a productive future object of study, while more discrete parcellation of other structures, like the hippocampus, striatum, orbital, and medial frontal cortex could provide greater insight into the evolutionary contributions of their functionally distinct subdivisions. Structures constituting the limbic system participate in diverse and distributed neural systems integrating emotion with sensory-motor and cognitive functions. Addressing differential volumetric change across these structures could provide a more complex and refined understanding of the evolutionary integrity of the limbic system and the importance of emotional behavior in human evolution.

### Conflict of interest statement

The authors declare that the research was conducted in the absence of any commercial or financial relationships that could be construed as a potential conflict of interest.
